# Disciplinary Research Ethics Cultures in Disaster Settings: Understandings from Researchers After 3.11 in Japan

**DOI:** 10.1177/15562646251399397

**Published:** 2025-12-08

**Authors:** Sudeepa Abeysinghe, Kaori Honda, Akihiko Ozaki, Alison Lloyd Williams, Claire Leppold, Aya Goto

**Affiliations:** 1 Global Health Policy Unit, School of Social and Political Science, 151027 University of Edinburgh, Edinburgh, UK; 2 Graduate School of Medicine, 13101 Tohoku University, Sendai, Japan; 3 469502 Jyoban Hospital of Tokiwa Foundation, Iwaki, Japan; 4 12775 Fukushima Medical University, Fukushima, Japan; 5 Department of Sociology, 151263 Lancaster University, Lancaster, UK; 6 Disaster, Climate & Adversity Unit, Melbourne School of Population and Global Health, University of Melbourne, Melbourne, Australia; 7 1857 Harvard T.H. Chan School of Public Health, Boston, USA

**Keywords:** Japan, 3.11, qualitative methods, research ethics, disciplinarity

## Abstract

Research ethics can be bound by implicit disciplinary understandings of what constitutes risk and how these should be mitigated in the field. The area of health research in post-disaster contexts draws in researchers from a variety of disciplines who are interested in a range of questions. In analysing researchers’ experiences of research ethics in the context of the 3.11 disaster in Japan, this paper investigates the ways in which disciplinary cultures of ethics are problematised and negotiated in this setting. It explores researchers’ perceptions of the ways in which different disciplines understand ethical practice, how these differences are illuminated in the context of working in close proximity with affected communities in a post-disaster setting, and how research ethics committees are negotiated in relation to disciplinary difference. The paper indicates some ways in which diverse cultures of research ethics might be better managed in future contexts.

## Introduction

There is increasing debate around what constitutes ethical research practice in post-disaster settings (Adams et al., 2024; Schobert et al., 2023). Despite this, there is a dearth of empirical work probing the perception of researchers themselves about their practice in such contexts (Abeysinghe & Leppold, 2023). Disaster settings present concentrated sites of research, involving an array of disciplines (Moezzi & Peek, 2021). Since disasters are characterised by both environmental and socio-political elements, disaster scholarship often requires researchers to extend their pre-existing expertise in order to meet the needs of affected communities (Abeysinghe et al., 2025; Donner & Diaz, 2017). A deeper understanding of the ways in which researchers negotiate research ethics in these contexts can potentially also provide insights into other settings where interdisciplinary and community-focused research occurs in a concentrated manner.

The 3.11 Fukushima ‘triple disaster’ presents an important case study in relation to the ethical practice of disaster research across disciplines. The disaster, which occurred in North-East Japan on March 11, 2011, is characterised by the interconnected elements of a 9.0 magnitude earthquake, an up to 15-meter tsunami impacting the North-East coastline, and radiation disaster surrounding the release of radioactive material from the Fukushima Daiichi powerplant, as well as the political and social responses to these. These intertwined aspects of 3.11 meant that medical, natural and social sciences are needed to fully understand the impacts (Few et al., 2022). This paper focuses on the accounts of researchers who work in the field of health in the context of 3.11; this includes medical professionals who engage in research, public health researchers, social scientists, humanities scholars and others. It focuses on the ways in which tensions and negotiations between disciplinary positions and structures of ethics play out, both in the field and in researchers’ engagement with the process of formal ethical review. This negotiation includes issues such as reaching a compromise or decision where disciplinary ethical norms within a team differ, choices around working with populations under stress and weighing potential stress caused by research against methodologically optimal research designs, reaching consensus when researchers come from different epistemological positions, and the management of expectations arising from ethics committees.

Medical research in Japan mandatorily undergoes formal ethical review through institutional research ethics committees (RECs). The first ethics committee for medical research involving human participants in Japan was founded at Tokushima University in 1982, with the number of such committees increasing steadily over time (Yanagawa et al., 2015). In 2006, the Japanese Ministry of Education required all universities in Japan to produce codes of ethical conduct for research (Macfarlane & Saitoh, 2008), with the Ministry of Health, Labour and Welfare subsequently implementing a system for certifying RECs in 2014 (Iijima et al., 2019). More recently, academic associations have started establishing ethical review committees for members who do not have a review mechanism at their institution. According to the national guidelines (MEXT, 2021), the committees should include: (i) a member who is an expert in a natural science; (ii) a member who is an expert in the humanities or social sciences; (iii) a member reflecting the opinions of the general public; (iv) at least two members who are external to the institution of the committee; (v) both male and female members, and; (vi) five or more members.

Within the field of medicine, RECs also exist outside the university setting in Japan. For example, many health researchers, especially in the case of the 3.11 disaster, are primarily employed in clinical work (Ozaki et al., 2020). In hospital settings, ethics committees assess ethical practice around both research (RECs) and clinical practice (hospital ethics committees – HECs) (Dowa et al., 2022). Typically, both hospital and university RECs in Japan are composed of a range of professionals, from clinicians to ethicists and lawyers (Sakaida et al., 2022). While engagement in these formal ethical structures is mandated for clinical research, use of formal ethics committees for other disciplines - such as within the humanities or social sciences of health - remains more sporadic; nonetheless, such fields have also developed disciplinary practices of research ethics in Japan (Togari et al., 2021). These disciplinary variations in expectation and practice can produce difficult terrain for interdisciplinary work. Within the context of interdisciplinary disaster research, as this paper shows, negotiations between the expectation of different disciplinary cultures of ethics are an important aspect of ethical practice.

## Methods

This study examines researcher perspectives on ethics within the interdisciplinary context of health and disaster research following the 3.11 disaster. It asks: how is ethics negotiated in the context of multidisciplinary health and disaster research? It draws upon semi-structured interviews with 14 key informants to reflect upon how interdisciplinary questions of ethics are understood and negotiated in this field.

### Sampling and Recruitment

We purposefully sampled for interviewees who represented a variety of expertise in health research following 3.11. This was undertaken in two steps: firstly, via a PubMed search to identify researchers who had published in the field and secondly, through snowballing and extending invitations through the authors’ research networks. This resultant sampling reflected diversity in relation to discipline, research methods, career stage and residence at the time of the disaster (i.e., a mixture of researchers who had already been working in Fukushima Prefecture and those who had come in to research the disaster setting). All of the interviewees were from Japan, and this study did not sample the international researchers that had been engaged in this setting, who bring with them additional ethics questions around cultural understanding of the site. (Table 1)

**Table 1.** Shows the Profiles of the Key Informants, with a Focus on Disciplinary Background and Research Methods. Disciplines and Methods are Described Broadly to Avoid Deanonymisation. the Number of Listed Methods Exceeds the Number of Interviewees Owing to Multi-Methods Researchers.

Characteristic	Participants
*Gender*	
Male	9
Female	5
*Discipline*	
STEM (science, technology, engineering, mathematics)	Including: medicine (2); nursing (2); clinical psychology (2); midwifery (1); health technician (1); engineering (1)
SHAPE (social science, humanities, arts, people, economy)	Including: anthropology (1); sociology (1); social psychology (1); legal studies (1)
*Methods*	
Quantitative and laboratory-based	Including: quantitative (8); physical field studies (2) experimental (1); sample analysis (1)
Qualitative	Including: qualitative (6); ethnography (1)
*Residence*	
In Fukushima Prefecture at the time of 3.11	4
Not in Fukushima Prefecture at the time of 3.11	10
*Career stage*	
Self-description of current career stage	Including: professor (5), lecturer (3), specially-appointed professor (2), assistant professor (2), principal investigator (1), early career researcher (1)

### Interviews and Analysis

The interviews opened with an explanation of the process and the collection of informed consent. Following this, the interviews probed several key themes in relation to both the formal and informal/practical exercise of research ethics. This paper focuses on reflections of formal ethics, and the way these were linked by interviewees to the issue of interdisciplinary research within this field site. Questions about researchers’ experience of the formal research ethics process were part of the interview schedule, while interdisciplinarity as a prominent theme emerged inductively through the responses of the interviewees themselves. The interviews were conducted by KH between December 2022-May 2023, and the majority of these (12 out of 14) were conducted online due to COVID-19. The interviews averaged 75 min in length.

Following transcription, the data was anonymised by KH and then translated into English. The data was checked by SA and AG. Subsequently, KH and SA independently undertook pilot coding via thematic analysis (Braun & Clarke, 2006). Following this, KH and SA compared approaches, and the formative results were also presented to a small group on interviewees as another form of validation, after which the coding was formalised and completed.

### Ethics Approval

This project was reviewed by the University of Edinburgh School of Social & Political Science Research Ethics Committee (application ID 284739) and received a favourable ethical opinion. Written informed consent was obtain, which was confirmed verbally at the point of interview.

## Results

In the context of interdisciplinary disaster research, researchers had to adapt their research ethics practices to take into account the context in which they were working. This included through expanding their understanding of research ethics (and methods) beyond the norms of their disciplines, understanding and engaging with the diverse disciplinary ethical cultures of their co-researchers, and effectively communicating with and engaging with RECs who sometime operate from a narrower or otherwise less suitable interpretation of research ethics.

### Stretching Disciplinary Ethical Norms

Following a major disaster, researchers can start to work in areas that extend beyond the reaches of their disciplinary expertise. In attending to the needs of the local population, researchers tend towards expanding the focus of research beyond disciplinarily-focused topics (Abeysinghe et al., 2025; Donner & Diaz, 2017), while health practitioners can become motivated to engage in community research following the experience of disaster (Abeysinghe et al., 2020). This can cause several complications in research ethics. In relation to 3.11, this included researchers working on topics (health), contexts (disaster, Tohoku region) or methods (e.g., moving from quantitative to qualitative, from environmental measurement to surveys with human participants) which were an extension from, or went beyond, the researchers’ previous methodological and ethical training. In particular, the interviewees suggested that most research happening in this context had a social element, but was often led by ‘people who are like newcomer social researchers’ (A) noting that ‘people who are qualified to be called social researchers must be very different, and there are ethics there, but [in this context] people who have never done it before can do it’ (A).

Researchers who had previously undertaken material-focused (e.g., chemical or environmental samples or measures) or quantitative health research moved into areas of research focusing on the community, deploying more qualitative methods in doing so. The practice and ethics of such qualitative research is often framed through quantitative epistemologies such that ‘… we tend to hear about qualitative research from the perspective of quantitative research’ (B). This resulted in a context where there was a mix of researchers that did not necessarily speak the same ethics ‘language’, including people drawing methodologically beyond their extant training.

As well as this, some researchers who had previously been working in non-health areas focused on health-related studies in the context of the disaster setting. This also presented challenges in that:

…there was a difference between people who were used to medical research and people who were not used to medical research, even if it was…by people who were experts in radiation, like physics people…people who have very good intentions, but who have never done medical research before, have come in and violated the [ethics] law and rules. (F)

As the quote above suggests, working outside presupposed disciplinary boundaries magnifies differences in ethical norms. The disaster context therefore saw pre-existing siloes between disciplines and methods rendered more permeable, and this had implications in terms of the consistency between how ethics was approached in this field and the ethical traditions of these disciplines.

### Disciplinary Cultures of Ethics

In response to this permeability of disciplines and approaches, the participants presented different accounts of the norms of ethical practices. Typically, the interviewees felt there were some groups of researchers who were operating with dissimilar understandings of research ethics. Moreover, there was variation amongst the interviewees about which disciplines were most likely to exercise good ethical conduct.

For example, a researcher from a science background noted the propensity for work in the social sciences and humanities to proceed without formal ethics review. The view that some social science and humanities disciplines practise in the disaster setting without formal ethical review was common:

…people in the humanities and sociology, who I personally believe think a lot about the protection of the research subjects, are skipping the ethics committee process. I wondered if they were thinking about it properly. There are quite a few sociologists who come in out of the blue, do interviews, record them and then write them down in a book. I thought that was an amazing [negatively] thing to do […] It is not just a matter of having an ethics committee, it is only a matter of process, so I think we should think about the way ethics in our field should be, and how it should be as a protection of human subjects. (C)

Where ethics review occurred, this is sometimes seen as inadequate, for example in the suggestion from some interviewees that (in Japan) anthropology ‘is quite relaxed about ethical review. So basically, there's not a lot of screening or anything like that before you do research, at the moment, not yet’ (N). This view is sometimes extended towards qualitative research in general where ‘there are a few people around me who are doing qualitative research in Fukushima, and they don't go through the ethics committee itself’ (C).

Conversely, critical reflections are also made towards natural scientists (by social and medical scientists), who are sometimes framed as misunderstanding the socio-political nature of conducting research in the site:

If you are doing something where people are not involved, like measuring the environment, then you probably don't need an ethics committee. That was their way of doing things, but this measurement of everyday life involves people, doesn't it? I remember persuading them that it [ethics review] would be necessary in that sense, because there would be people living there. (L)

As the extracts show, within this interdisciplinary research site scepticism around the ethical conduct of other disciplines comes to the fore as a site of reflection. However, while some interviewees locate this difference in individual practice, it is also clear that the structures and disciplinary cultures of ethical conduct undergird these patterns of engagement with formal ethics process. There is also a question of the difference between the attainment of formal ethical review and the conduct of ethical research practice, given that ‘even if there is no approval by an ethics committee, the researchers themselves can still follow the ethical aspects, right, the researchers themselves?’ (C).

In addition to the disciplinary differences, the values held by researchers who had been working in Fukushima before the disaster differed from those held by researchers who came after the disaster. The former emphasized avoiding harm caused by research, addressing residents’ anxiety, making informed consent beneficial, and preserving local voices, while the latter focused more on maintaining the objectivity of research and prioritizing local benefits.

### Research Ethics Committees

In the context of researchers working within distinct disciplinary ethical cultures, and simultaneously attempting to coordinate themselves in the multidisciplinary site of 3.11 disaster research, questions surrounding the inconsistencies of formal ethics processes come to the forefront.

Participants noted that ethics committees were not comprehensive across disciplines and institutions in Japan, while emphasising that ethical practice extends far beyond formal ethics review:

When you say you don't go through the ethics committee, it's because you don't have one, like [a specific] University. This is my opinion, but ethics committees are not a formality, and it is not just a matter of passing them, but basically from the perspective of protecting the subjects. I don't know if I can say this, but I felt that at [a certain] university, the ethics committee was designed to protect the researchers. […] and my current affiliation has a tendency to be like that, but it was originally intended to protect the subjects. (C)

Interviewees noted that ethical review can focus on administrative matters rather than engage with research practices on the case-by-case basis. For instance, this interviewee presents some discomfort over the idea that their research was (from their perspective) inadequately assessed:

… I had to do research that inevitably had to dig for traumatic things. I wrote that part with a lot of care… I double- and triple-prepared it, but nothing was said [by the REC] there. Rather, I was told that the data should be stored properly for a number of years, and so on. That part, I would do that, but that wouldn't be the essence of it, in the ethics review. […] I often think that they lack imagination in terms of the potential damage to informants in taking data and so on. (J)

This is sometimes linked to the idea that research ethics present a set of universal principles, and that RECs might not be attuned to the practical ethics of particular approaches or contexts:

Maybe one of the difficulties now is that one simple problem is that the scope of ethical review has become too broad. Every so-called university has to do it on its own…. But that is supposed to take care of everything from the review of ES cells for regenerative medicine to the review of sociological research. (J)

From this, researchers perceive that institutional ethics are often inconsistent in their approach to review, not attuned to the specifics of a research site or method, and are often experienced largely as administrative hurdles that are divorced from the actual practice of ethical research.

The practice of the Japanese RECs that fed into the 3.11 research was experienced as varied, such that researchers were concerned with the perceived ‘difficulty’ of their REC rather than the ethical complexity of the proposed research, underpinned by the understanding that ‘University ethics review committees vary from university to university and hospital to hospital, in terms of the level of approval for applications.’ (I)

These variations were seen as particularly pronounced in the case of research that is not commonly reviewed by RECs. While in the section above we show that social science and humanities disciplines are seen to infrequently undergo REC review, at the same time it is understood that RECs find the processing of ethics from these disciplines to be a challenge to their structures:

I have heard that qualitative research tended to have difficulty in being accepted by ethics committees, so I think that this may be a problem of the way ethics committees should be…. (C)

In some instances, the basic understanding of qualitative data collection as constituting ‘research’ has been experienced as contentious within formal ethics structures. Certain institutional approval points were also perceived as lacking in formal ethics infrastructure:

Specifically, municipalities don't have ethics committees, and environmental fields don't have ethics committees either. Also, the agricultural sector does not have an ethics committee, so I think this has been an issue in terms of ethics committees. (L)

In this way, the confluence of varied health-related disciplines in the 3.11 research site highlighted disciplinary ways of viewing research ethics, gaps in the ensured provision of ethical review, as well as the difficulties of negotiating ethical practice between groups of interdisciplinary researchers.

## Discussion

Research ethics are informed by disciplinary norms and practices, and in interdisciplinary contexts, these can become challenged. Following 3.11, health researchers expanded their methodological repertoire and were simultaneously challenged to work beyond their training in relation to research ethics. This observation leads to some practical recommendations in terms of thinking more holistically about research ethics in the context of disaster and health research, in relation to the work and composition of RECs and to the ethical thinking and practices of researchers.

Scholars and practitioners from varied disciplinary and methodological backgrounds were drawn to 3.11. Such a concentration of multidisciplinary research provides an opportunity to interrogate the ethics of research across disciplines. Few et al. (2022, p. 1) note that disaster research draws across and balances a diverse set of disciplines, resulting in collaborative engagement that is ‘likely to ask [researchers] to cross epistemological boundaries’.

In such contexts, tensions arise surrounding disciplinarily-bound ways of doing research. Research ethics are a key site at which disciplinary junctures become relevant, since they underpin the movement between disciplinary ways of knowing and ways of doing (Balsamo & Mitcham, 2010). As our interviewees attest, forms of practice that straddle disciplinary methods are particularly demanding. The formal organisational structures of research ethics tend to fall along established disciplinary boundaries (Forino et al., 2024). Here, the use of qualitative methods in the health field was challenging in the Japanese context where there is a strong focus on medical research ethics but less of a focus formally on ethics for non-medical disciplines. Even where health-related non-medical researchers seek to have their work examined by an institutional REC they find that, as Balsamo (2017, p. 255) suggests, these requests are not ‘easily accommodated within established institutions that govern and sanction knowledge production.’

This posed challenges for the researchers that we interviewed in multiple ways. Engaging with other disciplines can be made difficult due to the lack of common understanding and experience of both formal and informal ethics processes; this appears to persist despite the mandatory inclusion of interdisciplinary members in Japanese RECs (MEXT, 2021). In general, interdisciplinary research can be impeded by difficulties in establishing a common language and way of regarding the research (Gilligan, 2021), and this is demonstrated in relation to ethics as well. Researchers within these contexts develop practical reflexivity in engaging around these sensitive topics and with communities who have faced stress, while the formal structures of ethical review are not regarded as sufficient in helping to manage these complexities. Improving the formal ethical structures to better suit the needs of researchers and populations in these contexts would be a valuable exercise.

One way in which this context challenged ethical practice was through the extension of researchers into methods that they had previously not employed. This included, in particular, the widespread use of qualitative forms of research to engage with the local community. There is a significant literature indicating that, across countries, qualitative research projects can experience challenges during undergoing review when institutional RECs are set up to serve all disciplines ‘universally’ (Halkias, 2024; Musoba et al., 2014; Opsal et al., 2016). The 3.11 research site also saw natural scientists increasingly engage with questions of public health, but – from the perspective of the interview participants – without sharing awareness of the formal requirements of medical research ethics that are enforced in Japan.

While not the focus of this paper, the data also suggests that distinctions between research ‘insiders’ and ‘outsiders’ can impact ethical engagement (Miyazawa, 2018). Within our sample, researchers who had close community links tended to locate ethical practice as work that harmonises with the perspectives of the community, while external researchers were more likely to focus on perceived objectivity and robustness. Discussion on the Japanese culture of drawing a line between “uchi” as insiders and “soto” as outsiders has been well articulated by Kang using a typical Japanese house structure with an entrance hall where people take off their shoes when entering (Kang, 2021). The hall can be seen as a space to observe how uchi and soto interact. Using this metaphor, Fukushima can be seen as becoming an entrance hall for disaster research, where inside and outside researchers interacted. In disaster research, the interaction of insider and outsider perspectives may be important because it layers onto the discussion of disciplinarity that we explore here, and these intersections could be fruitfully interrogated further (cf. Goodall et al., 2022).

These findings lead to several practical suggestions around the conduct of research in contexts where there is a confluence of disciplinary research cultures. These are relevant to disaster settings, but also potentially to crisis, conflict and humanitarian crises, which can similarly draw diverse researchers. Some areas of reflection might include: ensuring the apparent gap between the formal mechanics of RECs (with the inclusion of interdisciplinary experts) is matched by the decisions and articulation of these decisions to researchers (to better signal an understanding of research beyond the medical/natural sciences); in contexts where there is a concentration of researchers, reflecting on the need for a local institutional ethics coordination point (O’Mathúna, 2010), such that institutions that do not regularly contend with complex ethics (e.g., agricultural research in the case of 3.11) have reviews supported by an experienced coordinating institution; researchers and their professional associations ensuring that formal ethical guidelines are understood and integrated into practice (Hastings et al., 2022), and developing a language of ethics that might aid in communicating and negotiating expectations with other disciplinary groups. These changes highlight the general trajectory of deepening ethics and integrity engagement in the Japanese research landscape (Kane et al., 2022; Suzuki & Sato, 2016).

### Best Practices

The gap between the formal interdisciplinary composition of RECs and their practical engagement with diverse methodologies should be addressed. Although Japan's national guidelines mandate interdisciplinary representation, this may not translate into informed review of non-biomedical approaches. Standardised training for REC members could help build shared understanding and improve review quality, particularly for qualitative and interdisciplinary research. In addition, cross-institutional ethics partnerships may help fill gaps where institutions lack experience reviewing certain methods or disciplines.

### Research Agenda

It would be useful to understand the extent to which less ethically-reflective individuals might negotiate the concerns described above, and the extent to which these issues are evident to such individuals. In general, there is a dearth of empirical evidence on the views of researchers about ethical practice. This is particularly the case in the field of disaster studies, despite the increasing interest in ethics as a topic within our field. Extending the empirical investigation to understand the extent to which these findings are replicated in other countries and settings would help determine the general applicability of these insights.

### Educational Implications

Further education of research communities on disciplinary differences in ethical practice would be useful, particularly given the increased focus on interdisciplinary research. This should start during the research training phase of research students.

### Limitations

This study engaged with purposive sampling to interview key informants around their experiences of research ethics following the 3.11 disaster. To some extent the participants were self-selecting; only researchers who had an interest in ethics were likely to respond to recruitment. This is not necessarily a limitation, in that the study was able to draw from the experiences of reflexive researchers. However, it is possible that if the sample included researchers who were less interested in speaking about research ethics some of the results may have differed. We recruited more STEM researchers than SHAPE researchers, and though this reflects the disciplinary balance at this field site, it is likely to have impacted on the reflections that we received. While the sample for this research is relatively small, this is a bounded community of researchers and the sample prioritised diversity.

## Conclusion

In settings where there is a sudden influx of researchers from different disciplines, the disciplinary cultures of ethics become more pronounced. In the context of 3.11 health and disaster research in Japan, researchers experienced tensions undergirded by varying disciplinary-bound expectations of ethical conduct. They also perceived differences in the availability, applicability, and operation of formal research ethics committees. Understanding and engaging with disciplinary cultures of ethics – at the formal level of research ethics committee, and more informally in relation to the interactions between researchers - can make it easier for researchers to negotiate these tensions and to ensure that ethical standards are widely recognised.
